# Treatment of Hypertrophy of the Heart

**Published:** 1845-09

**Authors:** 


*Treatment of Hypertrophy of the Heart.*—The author gives his opinion against the usual method of treatment by blood-letting in hypertrophy of the heart. The hypertrophy causes a disturbance of the bodily equilibrium, and the whole body becomes emaciated, whilst the functions and dimensions of the heart increase. The frame ought rather to be strengthened by a mild nourishing diet, and every excitement avoided. The inutility of fox-glove need not be expatiated upon, since it is generally acknowledged; nor do the combinations of iodine counteract the cardic hypertrophy; besides, they are frequently contraindicated by bronchial catarrh, cerebral congestion, &c. The author recommends mercury in the form of proto-ioduret (mercur. iodat. flavus) till severe salivation be induced. The patients must remain in bed, be well covered, and take diaphoretic drinks and purgatives. The fibrin of the blood is diminished by the above treatment, and the muscular tissue altered. The author never perceived vascular excitement ensue from the treatment he recommends, inproof of which he narrates five cases.—*Ibid, from Dr. Gottschall, of Cologne, in Caspar's Wochenshrift.*

